# Biallelic germline *BRCA1* mutations in a patient with early onset breast cancer, mild Fanconi anemia‐like phenotype, and no chromosome fragility

**DOI:** 10.1002/mgg3.863

**Published:** 2019-07-25

**Authors:** Katharina Keupp, Stephanie Hampp, Annette Hübbel, Monika Maringa, Sarah Kostezka, Kerstin Rhiem, Anke Waha, Barbara Wappenschmidt, Roser Pujol, Jordi Surrallés, Rita K. Schmutzler, Lisa Wiesmüller, Eric Hahnen

**Affiliations:** ^1^ Center for Familial Breast and Ovarian Cancer, Center for Integrated Oncology (CIO), University of Cologne, Faculty of Medicine and University Hospital Cologne, Cologne, Germany University Hospital of Cologne Cologne Germany; ^2^ Department of Obstetrics and Gynecology Ulm University Ulm Germany; ^3^ Department of Genetics and Biomedical Research Institute Hospital de Sant Pau Barcelona Spain; ^4^ Department of Genetics and Microbiology Universitat Autònoma de Barcelona Barcelona Spain; ^5^ Center for Biomedical Network Research on Rare Diseases Barcelona Spain

**Keywords:** biallelic *BRCA1*, early onset breast cancer, Fanconi anemia, p.Arg1699Gln

## Abstract

**Background:**

Biallelic *BRCA1* mutations are regarded either embryonically lethal or to cause Fanconi anemia (FA), a genomic instability syndrome characterized by bone marrow failure, developmental abnormalities, and cancer predisposition. We report biallelic *BRCA1* mutations c.181T > G (p.Cys61Gly) and c.5096G > A (p.Arg1699Gln) in a woman with breast cancer diagnosed at the age of 30 years. The common European founder mutation p.Cys61Gly confers high cancer risk, whereas the deleterious p.Arg1699Gln is hypomorphic and was suggested to confer intermediate cancer risk.

**Methods and Results:**

Aside from significant toxicity from chemotherapy, the patient showed mild FA‐like features (e.g., short stature, microcephaly, skin hyperpigmentation). Chromosome fragility, a hallmark of FA patient cells, was not present in patient‐derived peripheral blood lymphocytes. We demonstrated that the p.Arg1699Gln mutation impairs DNA double‐strand break repair, elevates RAD51 foci levels at baseline, and compromises BRCA1 protein function in protecting from replication stress. Although the p.Arg1699Gln mutation compromises BRCA1 function, the residual activity of the p.Arg1699Gln allele likely prevents from chromosome fragility and a more severe FA phenotype.

**Conclusion:**

Our data expand the clinical spectrum associated with biallelic *BRCA1* mutations, ranging from embryonic lethality to a mild FA‐like phenotype and no chromosome fragility.

## 1 INTRODUCTION

Monoallelic germline mutations in the *BRCA1* and *BRCA2* confer high life‐time risks for breast cancer (BC) and ovarian cancer (OC) (Kuchenbaecker et al., 2017) and were found in approximately 24% of index patients who met the inclusion criteria of the German Consortium for Hereditary Breast and Ovarian Cancer (GC‐HBOC) for germline testing (Kast et al., 2016). *BRCA1/BRCA2*‐related BC and OC susceptibility is inherited in an autosomal dominant manner with incomplete penetrance (Kuchenbaecker et al., 2017).

Biallelic (i.e., homozygous/compound heterozygous) mutations in the *BRCA2* cause Fanconi anemia (FANCD1, MIM# 605724), a recessive, congenital genomic instability syndrome generally characterized by bone marrow failure, developmental abnormalities in various organ systems and a high predisposition for hematological or solid tumors. Despite a similar mutation prevalence of the *BRCA2* and *BRCA1* in the general population (Maxwell, Domchek, Nathanson, & Robson, 2016), biallelic mutations affecting the *BRCA1* are rarely described and have been suggested as lethal during embryonic development (Goldgar et al., 2004), consistent with the embryonic lethality observed in *Brca1* null mice (Evers & Jonkers, 2006). In 2013, however, Domchek et al. described a 28‐year‐old woman with OC and an otherwise complex phenotype suggesting Fanconi anemia (FA). Medical records revealed short stature, microcephaly, developmental delay, and significant toxicity from chemotherapy. The patient carried a protein‐truncating variant (p.Asp821Ilefs*25) and a pathogenic missense variant (p.Val1736Ala) *in trans* (Domchek et al., 2013). In 2015, Sawyer et al. reported biallelic *BRCA1* mutations (p.Ser198Argfs*35 and p.Arg1699Trp) in a woman with multiple congenital anomalies consistent with a FA‐like disorder (phenotype subsequently referred to as FANCS, MIM# 617883) and BC at the age of 23 years (Sawyer et al., 2015). Freire et al. described a 2.5‐year‐old girl of consanguineous offspring with severe short stature, microcephaly, neurodevelopmental delay, congenital heart disease and dysmorphic features, and the girl was a homozygous carrier of a *BRCA1* nonsense mutation in exon 11 (p.Cys903*) (Freire et al., 2018). In 2018, Seo et al. reported multiple congenital anomalies and severe chromosomal fragility in children of consanguineous offspring, all homozygously carrying *BRCA1* nonsense mutations affecting exon 11 (p.Trp372*, p.Leu431*)(Seo et al., 2018). Of note, mitomycin C‐ or diepoxybutane (DEB)‐induced chromosome fragility assays performed by Seo et al. are routinely used to confirm clinical FA diagnosis (Castella et al., 2011).

In this study, we report a female index patient with two deleterious *BRCA1* missense alterations in a compound heterozygous state, namely, c.181T > G (p.Cys61Gly) and c.5096G > A (p.Arg1699Gln) (Bouwman et al., 2013; Lindor et al., 2012). The c.181T > G (p.Cys61Gly) alteration is one of the most common pathogenic *BRCA1* founder mutations in Europe (Rebbeck et al., 2018) and a well‐established high‐risk variant (Lindor et al., 2012), whereas the deleterious p.Arg1699Gln mutation is hypomorphic and was suggested to confer intermediate cancer risk (Bouwman et al., 2013; Moghadasi et al., 2018; Spurdle et al., 2012).

## 2 MATERIALS AND METHODS

### 2.1 Testing for germline mutations in cancer predisposition genes

**Figure 1:**
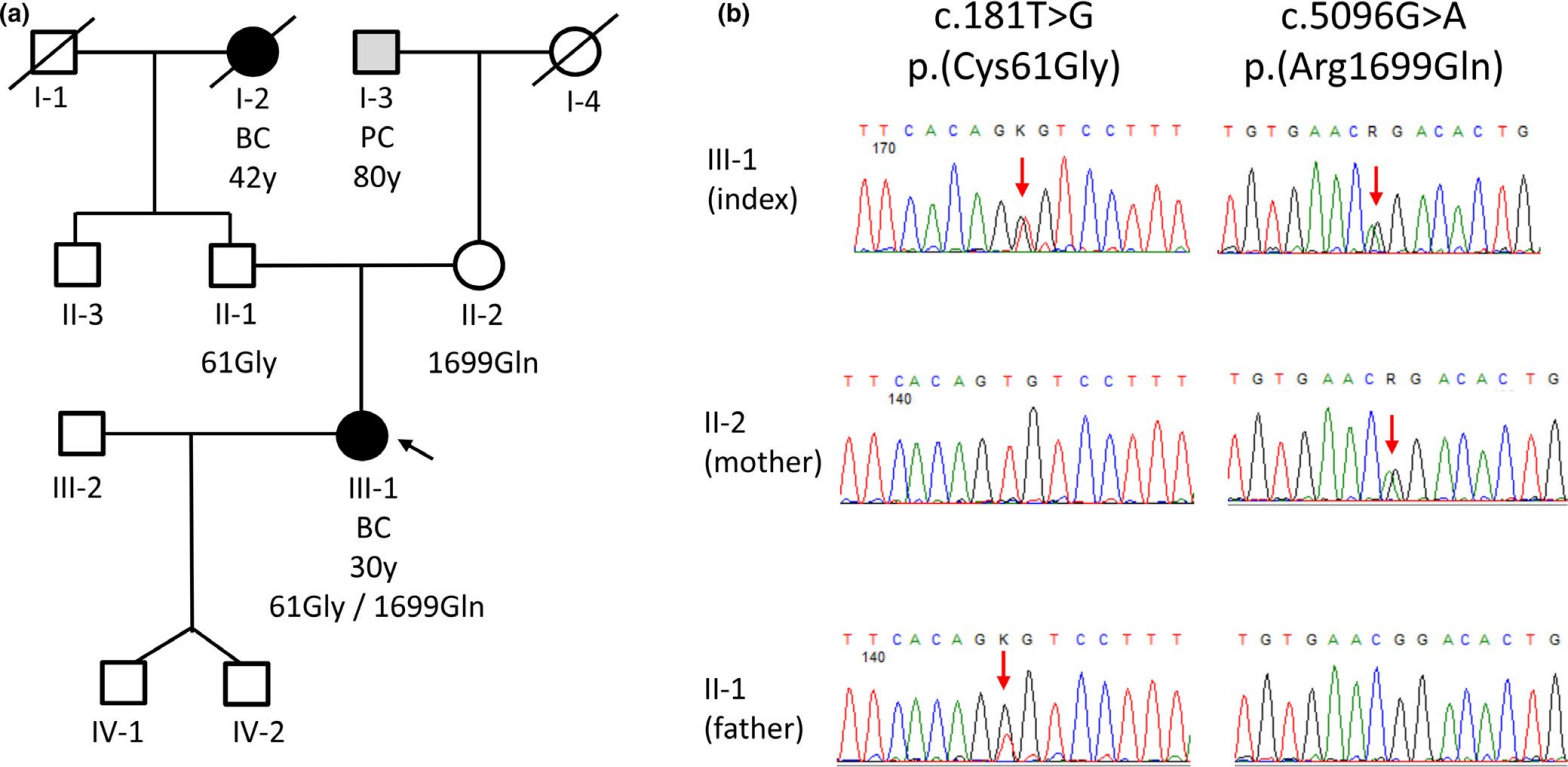
Pedigree and germline mutation status. (a) Cancer family history of the index patient (III‐1, marked with an arrow), including age at first diagnosis. BC = breast cancer; PC = prostate cancer; y = years. The index patient's mother (II‐2) had no personal history of cancer at the age of 60 years. The maternal grandfather of the index patient was diagnosed with prostate cancer at the age of 80 years. The index patient's father (II‐1) had no personal history of cancer at the age of 59 years. The paternal grandmother of the index patient was diagnosed with breast cancer at the age of 42 years and died at the age of 87 years. No other cancer cases were reported in the family. (b) Sanger sequencing electropherograms show *BRCA1* genotypes (*BRCA1* reference transcript NM_007294.3) of the index patient III‐1 and both parents (II‐1, II‐2). Red arrows mark the presence of a single nucleotide substitution

The index patient III‐1 and her parents (Figure 1a) were counseled at the Center for Hereditary Breast and Ovarian Cancer, University Hospital of Cologne, Germany. Physicians qualified in genetic counseling recorded personal and family cancer history, information regarding age at first diagnosis, tumor receptor status, and treatment of the index patient. Written informed consent was obtained from all individuals, and ethical approval was granted by the ethics committee of the University of Cologne (07‐048). Genomic DNA was isolated from venous blood samples. Next generation sequencing technology was applied using an Illumina MiSeq sequencing device (Illumina, San Diego, USA) and the customized diagnostic TruRisk® multigene panel for target enrichment (Agilent, Santa Clara, USA) covering *BRCA1*, *BRCA2*, and additional breast/ovarian cancer predisposition genes (*ATM, BRIP1, CDH1, CHEK2, PALB2, RAD51C, RAD51D, TP53*). Variant classification was performed in accordance with the regulations of the international ENIGMA consortium (Evidence‐based Network for the Interpretation of Germline Mutant Alleles; https://enigmaconsortium.org; version 1.1:26 March 2015).

### 2.2 Blood‐derived cell cultures for functional analyses

The DEB‐induced chromosome fragility assay was performed as described previously (Castella et al., 2011). PBLs were partially isolated from heparinized blood samples by Ficoll‐Paque‐PLUS, (LSM 1077 Lymphocyte, GE Healthcare, Germany) gradient centrifugation followed by several washing steps with PBS to remove thrombocytes, and the samples were then gently frozen as described (Deniz, 2017). The remaining fractions of the blood samples from the index patient (III‐1, Figure 1a) and her mother (II‐2, Figure 1a) were subjected to the establishment of LCLs by EBV‐immortalization of peripheral blood mononuclear cells. Aliquots from two reference blood samples were obtained from healthy female donors aged 28 and 26 (approval #157/2010 and amendment 2016 by the ethics committee of Ulm University), from which aliquots of the same blood draw had previously served as references in a recent case–control study (Deniz, 2017). LCLs from three reference individuals age 49–56 were used as controls during measurements of microhomology‐mediated end joining (Figure S1a). Thawed PBLs were cultivated in PB‐MAXTM Karyotyping Medium (Gibco/Invitrogen, Carlsbad, CA, USA) including 2% phytohemagglutinin (PAA, Pasching, Germany) for 72 hr. LCLs were cultivated as previously described (Gatz et al., 2016) and used for HR measurements at passage 2–6.

### 2.3 EGFP‐based reporter for DSB repair measurements

**Figure 2:**
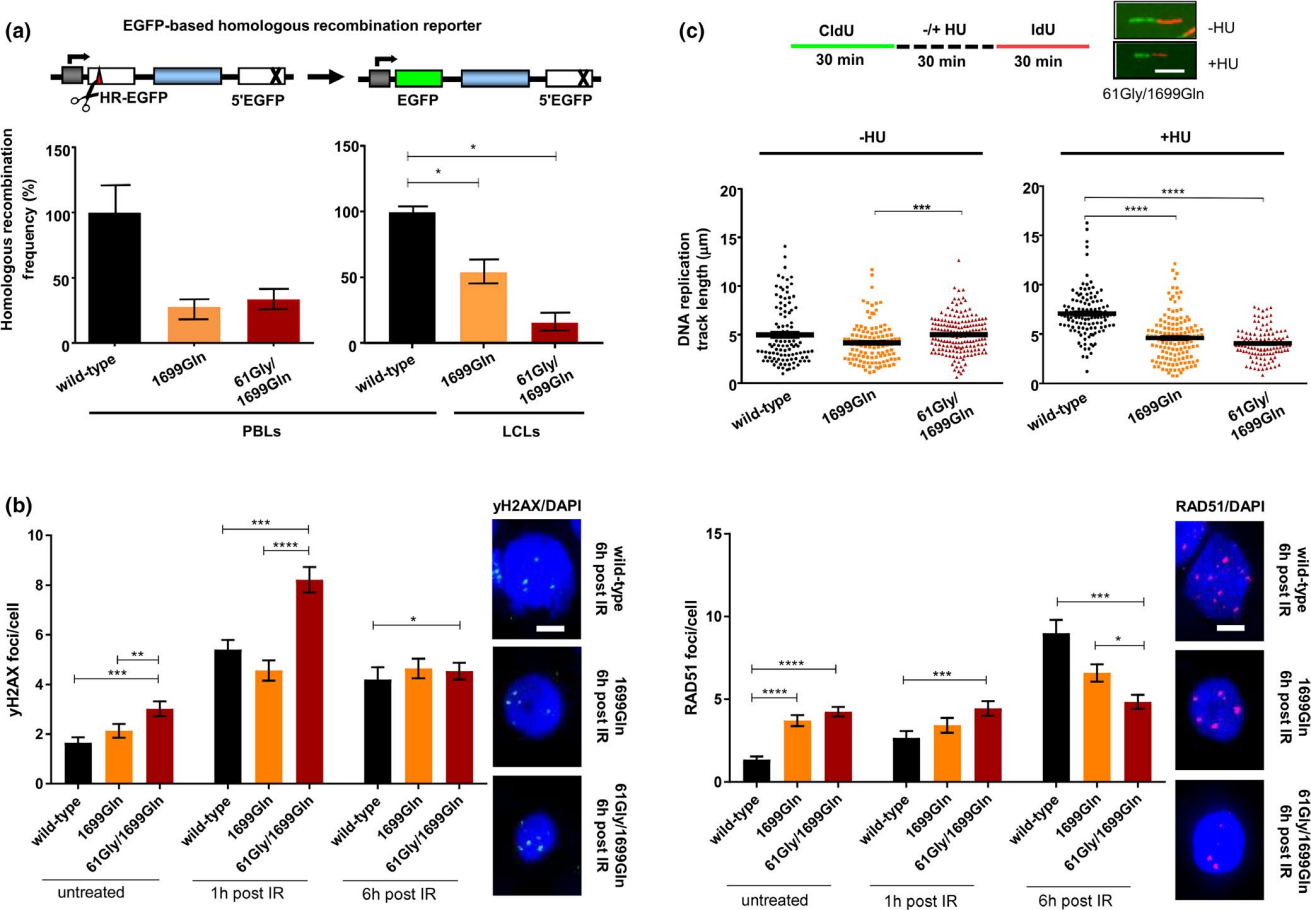
(a) Analysis of DSB‐induced HR activities in individuals with wild‐type, mono‐ or biallelic mutations in the *BRCA1*. To measure the repair of DSBs by HR, we used the EGFP‐based reporter substrate HR‐EGFP/5'EGFP consisting of mutated EGFP genes with an I‐*Sce*I endonuclease recognition sequence replacing 4 bp (HR‐EGFP) and a 3' truncation (5'EGFP), respectively. The DSB repair substrate for the determination of HR frequencies is schematically drawn on top (Akyuz et al., 2002). I‐*Sce*I recognition sequence, red triangle; cross, truncating mutation; white bars, mutated *EGFP* genes; dark green bars, reconstituted *EGFP*; blue bar, spacer sequence; gray bar with kinked arrow, transcriptional promoter; scissors, I‐*Sce*I endonuclease. HR measurements were performed 24 hr following transfection of PBLs (left diagram) or LCLs (right diagram) from individuals with BRCA1 (p.Arg1699Gln) or BRCA1 (p.Cys61Gly/p.Arg1699Gln) with HR‐EGFP/5´EGFP substrate plus I‐*Sce*I expression plasmid for substrate cleavage. Measurements for PBLs with wild‐type BRCA1 were obtained from one (left diagram) and two (right diagram) independent control individuals. Percentages of EGFP‐positive live cells were normalized to the individually determined transfection efficiencies for HR frequency calculations. Mean values of wild‐type controls were set to 100% (absolute mean frequency: 0.2%). Mean values and standard errors of the mean (*SEM*) from 3 to 5 measurements are shown. (b) Immunofluorescence microscopic analysis of DNA damage and RAD51 nucleofilament formation. Nuclear foci of the DNA damage marker γH2AX (left diagram) and of RAD51 (right diagram), indicating nucleofilament formation, were analyzed in PBLs from individuals with wild‐type and mutated BRCA1 (p.Arg1699Gln, p.Cys61Gly/p.Arg1699Gln) 1 and 6 hr after treatment with 2 Gy ionizing radiation (IR) and in untreated samples. Immunolabeled foci were scored by automated quantification from 97 to 115 nuclei per sample. Mean foci/cell and *SEM* are graphically shown on the left side. Statistically significant differences were determined using nonparametric Mann–Whitney test for unpaired samples with the software GraphPad Prism version 7.03. *p < .05; **p < .01; ***p < .001; ****p < .0001. Representative images of green γH2AX and red RAD51 foci in DAPI‐stained nuclei (blue) 6 hr post‐IR are shown on the right side. The size bar indicates 5 µm. (c) Analysis of replication fork protection following stalling. Single DNA fiber analysis was performed as schematically outlined in the sketch on top. Representative fibers ∓ HU are shown for the patient carrying biallelic BRCA1 mutations (p.Cys61Gly/p.Arg1699Gln). The size bar indicates 5 µm. CldU track length distributions of CldU‐ and IdU‐positive DNA fibers from PBLs derived from individuals with wild‐type and mutated BRCA1 (p.Arg1699Gln, p.Cys61Gly/p.Arg1699Gln) were determined in the presence of HU, indicating replication stalling (right) or absence of HU for unperturbed replication. Mean values were calculated from fiber track lengths of 106–172 single fibers in each sample. Statistically significant differences between PBLs from individuals with wild‐type and mutated BRCA1 (p.Cys61Gly/p.Arg1699Gln) were calculated using Dunn's test. ***p < .001; ****p < .0001

To analyze DSB repair via HR or microhomology‐mediated end joining, we used our enhanced green fluorescent protein (EGFP)‐based test system, as described earlier (Akyuz et al., 2002; Deniz, 2017; Obermeier et al., 2016). Briefly, the expression plasmid for the endonuclease I‐*Sce*I (pCMV‐I‐SceI) together with HR or microhomology‐mediated end joining substrate (Figure 2a and Supp. Figure S1a) and filler plasmid (pBlueScriptII KS; Stratagene, Heidelberg, Germany) or wild‐type EGFP reporter plasmid (for determination of transfection efficiencies) were transfected into PBLs or LCLs using amaxa reagents (Lonza, Cologne, Germany). After 24 hr of cultivation, reconstitution of wild‐type EGFP was determined by FACS analysis of the fraction of green fluorescent cells among living cells identified by gating in the FL1/FL2 and side scatter/forward scatter dot plots following laser excitation at 488 nm (FACS Calibur FACScan, Becton Dickinson, Heidelberg, Germany). Each quantification of green fluorescent cells monitoring DSB repair was normalized using the individually determined transfection efficiency (20%–60%) to calculate the DSB repair frequency.

### 2.4 Nuclear foci analysis by immunofluorescence microscopy

For in situ analysis of DSB repair by HR, we performed immunofluorescence microscopic analysis of the DNA damage marker γH2AX and the recombinase RAD51 in PBLs after exposure to 2 Gy of ionizing radiation (IR) as previously described (Gatz et al., 2016; Obermeier et al., 2016). Briefly, PBLs were harvested by cytospinning at the indicated time points postirradiation, fixed with 3.7% formaldehyde followed by permeabilization with 0.5% TritionX‐100. Primary antibodies were directed against γH2AX (Ser139, clone JBW301, Millipore, Billerica, MA, USA) and RAD51 (H‐92, Santa Cruz Biotechnology, Heidelberg, Germany), and the secondary antibodies were AlexaFluor488 and 555‐labeled (Invitrogen, Karlsruhe, Germany). Immunostained cells were mounted with VectaShield mounting media containing DAPI (Vector laboratories, Burlingame, CA, USA). Focal accumulations of 53BP1 and RAD51 in ≥97 DAPI‐stained nuclei from two independent slides were analyzed using a Keyence BZ‐9000 microscope equipped with Keyence BZ‐II Analyzer software (Keyence, Neu‐Isenburg, Germany).

### 2.5 Cellular sensitivity to PARP inhibition and carboplatin

To assess the response to the PARP inhibitor olaparib (Biozol, Eching, Germany), PBLs were treated with various olaparib concentrations (0.5–512 μmol/L) or carboplatin (0.125–2048 µmol/L) for 24 hr and subsequently re‐cultivated in fresh medium for 24 hr. Cell viabilities after the different treatments were determined in duplicate using the 3‐(4,5‐dimethylthiazol‐2‐yl)‐2,5‐diphenyltetrazolium bromide (MTT) assay as described (Deniz, 2017).

### 2.6 DNA fiber assay for nascent DNA synthesis

Cells were labeled with 20 µmol/L 5‐chloro‐2‐deoxyuridine (CldU, Sigma‐Aldrich, Steinheim, Germany) for 30 min and centrifuged for 2 min to discard CldU‐containing medium before treating the cells with 5 mmol/L hydroxyurea (HU, Sigma‐Aldrich, Steinheim, Germany) for 30 min or mock‐treated. To discard HU‐containing medium, the cells were centrifuged for 2 min, then, the cells were incubated with 200 µmol/L 5‐Iodo‐2 deoxyuridine (IdU, Sigma‐Aldrich, Steinheim, Germany) for 30 min. For cell harvesting, the cells were centrifuged for 2 min and washed once with 1 × DPBS before the cell pellet was re‐suspended in 1 × DPBS. Cell‐spotting, cell lysis, fixation and immunofluorescence staining were performed as previously described (Hampp et al., 2016). Briefly, the cells were spotted on a slide, and the DNA spreading was performed via gravity before fixation in methanol/acetic acid. For denaturation/depurination, glass slides were incubated in HCl before immunofluorescence staining.

### 2.7 Statistical analyses

Statistically significant differences between mean values of DSB repair frequencies or nuclear foci/cell numbers were calculated using the Mann–Whitney test for unpaired samples with the software GraphPad Prism version 7.03. GraphPadPrism 7.03 was also used for calculating IC_50_ values and to test for significant differences between olaparib‐response curves using the extra sum‐of‐squares *F*‐test. Kruskal–Wallis based Dunn's multiple comparison test was used for the calculation of statistically significant differences in nascent DNA synthesis. All *p‐*values are two‐sided with *p* < .05 considered statistically significant.

## 3 RESULTS

### 3.1 Clinical findings

The female index patient presented at the age of 30 years with an invasive‐ductal carcinoma of her left breast (G3; Ki67 30%). The 35‐mm tumor expressed estrogen receptors but tested negative for progesterone receptor and epidermal growth factor receptor 2 expression/overexpression. At the time of the BC diagnosis, the patient's blood count was inconspicuous. During her childhood, she obtained diagnoses of hearing loss (right side), celiac disease and congenital hip dislocation (left side) and had a hip operation at 11 years. A dysmorphology exam revealed a short height (150 cm, <5%ile for adult height) (de Onis et al., 2007), microcephaly (52 cm, <5%ile for adult head circumference), triangular face with low placed ears, and skin hyperpigmentation (several café au lait macules). No further FA‐like features were observed (no limb defects; no ophthalmic, endocrine, genitourinary tract, spine or neck anomalies; no congenital heart defect; no gastrointestinal malformations; no central nervous system anomalities). Her parent's heights were unconspicuous (mother: 179 cm, father: 185 cm). Following BC diagnosis, she received neoadjuvant chemotherapy starting with four cycles epirubicin/cyclophosphamide (90/600 mg/m^2^ body surface). Doses were reduced during the fourth cycle due to hematotoxicity (75/400 mg/m^2^ body surface). She subsequently was treated with paclitaxel (80 mg/m^2^ body surface), which was combined with carboplatin (AUC 5) starting from the second cycle. Due to recurring thrombopenia, neutropenia, and anemia, she received reduced doses of paclitaxel (70 mg/m^2^ body surface) and carboplatin (AUC 1.5). As a result of the prolonged thrombopenia, neutropenia, and anemia, chemotherapy was discontinued after four cycles, followed by mastectomy, and radiotherapy and antihormone therapy.

### 3.2 Cancer family history and germline mutation status

The pedigree of the index patient III‐1 is shown in Figure 1a. The index patient met the inclusion criteria of the German Consortium for Hereditary Breast and Ovarian Cancer (GC‐HBOC) for germline testing, which was performed by next generation sequencing (NGS) using the TruRisk® gene panel. Two heterozygous missense mutations, c.5096G > A (p.Arg1699Gln) and c.181T > G (p.Cys61Gly), in the *BRCA1* were detected, which both were confirmed by Sanger sequencing (Figure 1b). Genetic testing of the patient's parents via Sanger sequencing revealed that the c.5096G > A (p.Arg1699Gln) mutation was inherited from the patient's mother (II‐2), whereas the c.181T > G (p.Cys61Gly) mutation was inherited from the patient's father (II‐1).

### 3.3 Diepoxybutane (DEB)‐induced chromosome fragility assay

Chromosome fragility is usually reported as “percentage of aberrant cells” and “breaks/cell.” The number of “breaks/cell” is more than 10 times higher in the FA population than in the non‐FA population, while the “percentage of aberrant cells” is increased 60 times in FA patients (Castella et al., 2011). To determine whether a FA phenotype may be present on the cellular level using peripheral blood lymphocytes (PBLs) derived from the index patient (III‐1) and her unaffected mother (II‐2), we used an established DEB‐induced chromosome fragility assay described previously (Castella et al., 2011). Both the index patient (6% of cells with breaks, 0.12 breaks/cell) and her unaffected mother (10% of cells with breaks, 0.17 breaks/cell) showed DEB‐induced chromosome fragility within the control range (4% of cells with breaks, range 0%–22%; 0.05 breaks/cell, range 0–0.5). These inconspicuous results prompted us to investigate the hypomorphic p.Arg1699Gln allele, which was suggested to confer intermediate cancer risk (Bouwman et al., 2013; Moghadasi et al., 2018; Spurdle et al., 2012), in more detail. The BRCA1 BRCT domain p.Arg1699Gln mutation in the index patient III‐1 and her mother II‐2 (Figure 1a) was described to weaken BACH1 phospho‐peptide interactions (Clapperton et al., 2004), which are required for efficient error‐free homologous recombination (HR) repair (Xie et al., 2010).

### 3.4 EGFP‐based reporter for DSB repair measurements

Given the close association between HR deficiency and the pathogenicity of *BRCA1* mutations (Bouwman et al., 2013), we applied an EGFP‐based DNA double‐strand break (DSB) repair assay on fresh PBLs derived from the index patient, her unaffected mother and two female healthy donors functionally representing wild‐type controls (Deniz, 2017; Gatz et al., 2016; Keimling et al., 2012) (Figure 2a). HR analysis indicated threefold reduced HR frequencies in PBLs from the patient as well as from the mother compared to the wild‐type reference, although not reaching statistical significance (*p* = .1000), most likely due to the limited number of measurements. This observation was supported in a second experiment using early passage lymphoblastoid cell lines (LCLs) derived from the same blood draws of the patient and her mother. Compared to the mean values for wild‐type *BRCA1* status (obtained with two wild‐type PBL controls), a statistically significant twofold decrease in HR was scored for the LCLs from the mother carrying the *BRCA1* p.Arg1699Gln mutation and sixfold for the LCLs from the index patient carrying both mutations, p.Cys61Gly and p.Arg1699Gln (*p* = .0357). Concomitantly, both LCLs showed four‐ to fivefold elevated microhomology‐mediated end joining compared with three wild‐type LCLs (*p* = .0182–.0091; Figure S1). These results indicated a defect in the HR machinery of the mother and the index patient, in particular, causing a derepression of error‐prone DSB repair. Notably, error‐prone DSB repair, such as microhomology‐mediated end joining, associates with BC/OC risk as previously shown for cells from *BRCA1*, *BRCA2*, and *PALB2* mutation carriers as well as in a case–control format independently of the individual risk genotype (Keimling et al., 2012, 2011; Obermeier et al., 2016).

### 3.5 Nuclear foci analyses (γH2AX and RAD51) by immunofluorescence microscopy

To elucidate the molecular defect observed, we performed immunofluorescence microscopic analysis of focal nuclear structures indicative of the accumulation and/or removal of DSBs using antibodies against γH2AX and of the assembly of the HR machinery using antibodies against RAD51. Prior to treatment, we observed 1.4‐fold elevated basal γH2AX foci levels in patient PBLs compared to PBLs from the mother and 1.8‐fold compared to the wild‐type control (Figure 2b). Moreover, basal RAD51 foci numbers were threefold higher in PBLs from the patient and mother than those in the control (Figure 2b). Because γH2AX foci were reported to mark not only DSBs but also other DNA lesions (Gagou, Zuazua‐Villar, & Meuth, 2010), and in particular, stalled replication forks triggering recombinative lesion bypass, our observation was consistent with pronounced replication stress marked by both γH2AX and RAD51 in patient cells. One hour after DSB induction by treatment of PBLs with 2 Gy ionizing radiation, γH2AX foci accumulated two‐ to threefold in all cell types (*p* < .001), thereby maintaining a relative twofold increment of γH2AX foci in patient cells (Figure 2b). At the same time point, RAD51 foci numbers still showed a similar pattern as before irradiation, because canonical nonhomologous end joining dominates during this early period of DSB repair (Deniz, 2017) (Figure 2b). However, 6 hr after irradiation, RAD51 foci were 3.4‐fold and 1.9‐fold elevated in cells from the control and mother, respectively (*p* < .001), when compared with 1 hr postirradiation (Figure 2b). No increase was observed in the patient cells suggesting a problem in RAD51 filament assembly during the late phase of DSB repair involving end processing‐mediated pathways such as HR (Deniz, 2017). Since γH2AX‐labeled DSB scores were similar in the different PBL cultures at this late time point (Figure 2b), DSB repair in patient cells may have shifted towards mutagenic pathways. Microhomology‐mediated end joining was reported to become unleashed in HR defective cells (Keimling et al., 2011) as demonstrated for the patient cells investigated here (Supp. Figure S1). Altogether, these data suggested that the acquisition of the *BRCA1* variant causing the p.Cys61Gly mutation on top of p.Arg1699Gln exchange severely altered HR in the patient, resulting in accumulation of basal DNA damage and failure to assemble functional RAD51 nucleofilaments.

### 3.6 Cellular sensitivity to Poly(ADP‐ribose) polymerase (PARP) inhibition and carboplatin

PARP inhibition causes synthetic lethality in combination with severe HR deficiency such as of tumor cells from *BRCA1* or *BRCA2* mutation carriers upon inactivation of the wild‐type allele (Bryant et al., 2005). Having identified increasing defectiveness in HR in the biallelically versus monoallelically *BRCA1* mutated and wild‐type cells, we examined cell survival after exposure to increasing concentrations of the PARP inhibitor olaparib. Patient cells were highly sensitive to olaparib treatment as compared to the control and to cells from the mother, for which we determined an IC_50_ value showing only a trend towards a significant decrease compared with the control (*p* < .1) (Supp. Table S1). Conversely, after treatment with the DNA cross‐linking agent carboplatin, we found no statistically significant difference between the IC_50_ values for the cells from the patient, mother or control (Table S1); although in parallel, we measured sensitivity changes in PBLs from a cohort of high BC/OC risk individuals (Deniz, 2017). These discrepant results could be because of DNA interstrand cross‐links, as discovered recently (Semlow, Zhang, Budzowska, Drohat, & Walter, 2016), and can be repaired by both HR‐dependent and ‐independent pathways.

### 3.7 DNA fiber assay for nascent DNA synthesis

Schlacher, Wu, & Jasin (2012) demonstrated that BRCA2 exerts functions in stabilizing DNA replication forks that can be separated from its role in promoting HR. Livingston and co‐workers (Pathania et al., 2014) observed that BRCA1 is haploinsufficient for the suppression of replication stress but not for RAD51 foci formation or PARP inhibitor sensitivity. To investigate the response of patient cells in the presence of replication stress, we analyzed the fate of nascent DNA replication tracks after treatment with hydroxyurea (HU), which causes the depletion of the deoxyribonucleotide pool (Figure 2c). In the absence of HU, replication track lengths were similar for PBLs from the mother or patient compared with the control (Figure 2c). However, tracks of PBLs from the mother and the patient were 30% and 40%, respectively, shorter after HU treatment compared to the control after HU treatment. Taken together, cells from both the mother carrying the p.Arg1699Gln mutation and the patient with both the p.Cys61Gly and the p.Arg1699Gln mutations are compromised in protecting nascent DNA from nucleolytic degradation during replication stress, whereas defective RAD51 foci formation postirradiation and olaparib hypersensitivity was observed with patient cells only.

## 4 DISCUSSION

We show the co‐occurrence of two deleterious *BRCA1* alterations (p.Cys61Gly, p.Arg1699Gln) *in trans* with no cytogenetic features of FA observed, indicating that at least one mutation is hypomorphic. The inconspicuous cytogenetic and comparatively mild FA‐like clinical phenotype presented here is most likely due to incomplete impairment of *BRCA1* function by the p.Arg1699Gln risk allele, which was described to intermediately increase BC/OC risks by the age of 70 years to 20% and 6%, respectively (Moghadasi et al., 2018; Spurdle et al., 2012). These calculated risks were considerably lower than those described for pathogenic mutations overall, which were associated with BC/OC risks by the age of 70 years of approximately 60% and 40%, respectively (Kuchenbaecker et al., 2017). Using PBLs/LCLs derived from the patient's unaffected mother, we demonstrated that the p.Arg1699Gln mutation impairs DSB repair causing a derepression of error‐prone DSB repair (Figure 2a and Figure S1), elevates RAD51 foci levels at baseline (Figure 2b), and compromises BRCA1 protein function in protecting from replication stress (Figure 2c). Although the p.Arg1699Gln mutation impairs DNA double‐strand break repair and compromises BRCA1 function in protection from replication stress, the residual function of the p.Arg1699Gln allele likely prevents from chromosome fragility and a more severe FA phenotype. In addition, we cannot exclude a residual function of the BRCA1 protein carrying the p.Cys61Gly mutation.

In concert with published findings (Domchek et al., 2013; Freire et al., 2018; Sawyer et al., 2015; Seo et al., 2018), our data suggest that the phenotypic variability of biallelic *BRCA1* mutations ranges from embryonic lethality to short body height, microcephaly, early onset BC and toxicity from chemotherapy, whereas residual BRCA1 protein function determines the disease severity. For FA patients homozygously carrying nonsense mutations in exon 11 of the *BRCA1* (Freire et al., 2018; Seo et al., 2018), it has been demonstrated that a naturally occurring alternative splicing isoform can enable survival, albeit with severe consequences (Seo et al., 2018). The index patient described by Sawyer et al. carried a protein‐truncating *BRCA1* mutation, p.Ser198Argfs*35, along with the p.Arg1699Trp high‐risk variant which was shown to elevate the BC risk to 58% by the age of 70 years (Spurdle et al., 2012). Analyses of patient‐derived cells revealed defective RAD51 foci formation and olaparib hypersensitivity, similar to the patient characterized in our study. In contrast, however, the FANCS patient described by Sawyer et al. showed DEB‐caused cross‐linker–induced chromosomal aberrations, a hallmark of FA patient cells, which was not present in our patient.

## CONFLICT OF INTEREST

The authors declare that they have no competing interests.
