# Supplementary material for: Biallelic germline BRCA1 mutations in a patient with early onset breast cancer, mild Fanconi anemia‐like phenotype, and no chromosome fragility
Source: Mol Genet Genomic Med. 2019 Jul 25;7(9):e863. doi: 10.1002/mgg3.863 (PMC6732317; doi:10.1002/mgg3.863)
Supplement: Supplementary file 1 [file MGG3-7-e863-s001.docx]

**Supplementary Table and Figure**

Biallelic germline BRCA1 mutations in a patient with early-onset breast cancer, mild Fanconi anemea-like phenotype and no chromosome fragility

| individual | *BRCA1* status | treatment | | | |
| --- | --- | --- | --- | --- | --- |
|  |  | olaparib | | carboplatin | |
|  |  | IC_50_ (μM) | *P* value | IC_50_ (μM) | *P* value |
| healthy control | wild-type | 194 | - | 36 | - |
| mother (II-2) | p.Arg1699Gln | 64 | 0.0942 | 44 | 0.6556 |
| index patient (III-1) | p.Cys61Gly  p.Arg1699Gln | 32 | 0.0086 | 25 | 0.1192 |

**Table S1: Cell viability of PBLs with wild-type versus mutated *BRCA1* gene.** Cell viabilities were determined using MTT assay after treatment with the PARP inhibitor olaparib or carboplatin at increasing drug concentrations (0.5-512 μM and 0.125-2048 µM, respectively) for 24 h and subsequent growth in fresh medium for another 24 h. IC_50_ values (μM) were determined from a survival curve automatically fitted by GraphPad Prism version 7.03. Statistically significant differences were calculated for differences between IC_50_ values of *BRCA1*-mutated versus wild-type reference PBLs using GraphPad Prism version 7.03.

**
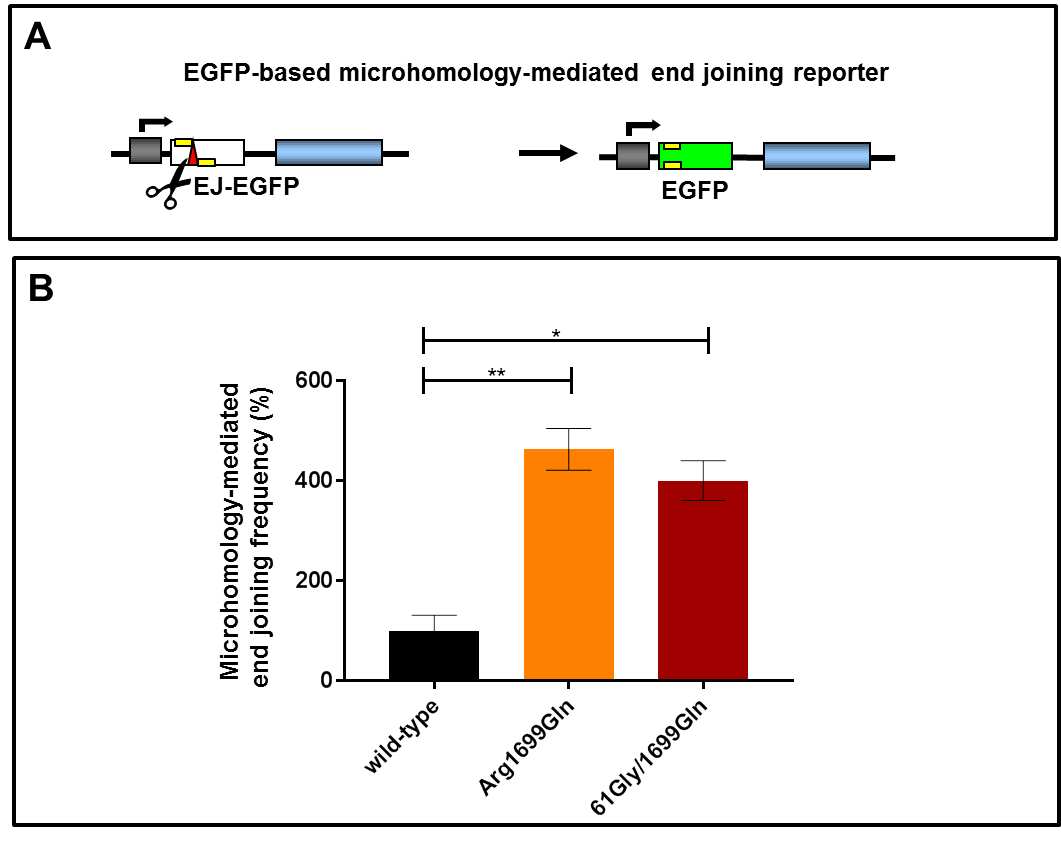
Figure S1. Analysis of microhomology-mediated end joining activities in individuals with wild-type, mono- or bi-allelic mutations in the *BRCA1* gene.** To measure the repair of DSBs by microhomology-mediated end joining, we used the EGFP-based reporter EJ-EGFP comprising mutated EGFP with an I-*Sce*I endonuclease recognition sequence flanked by 5 bp microhomologies. (**A**) The DSB repair substrate for the determination of homologous recombination frequencies is schematically drawn on top [29]. I-*Sce*I recognition sequence, red triangle; cross, truncating mutation; white bars, mutated EGFP genes; dark green bars, reconstituted EGFP; blue bar, spacer sequence; gray bar with kinked arrow, transcriptional promoter; scissors, I-*Sce*I endonuclease. (**B**) Microhomology-mediated end joining measurements were performed 24 h following the transfection of LCLs derived from three wild-type controls, or from individuals with BRCA1 (p.Arg1699Gln) or BRCA1 (p.Cys61Gly/p.Arg1699Gln), with EJ-EGFP substrate plus the I-*Sce*I expression plasmid for substrate cleavage. Percentages of EGFP-positive live cells were normalized to the individually determined transfection efficiencies for microhomology-mediated end joining frequency calculations. Mean values of wild-type controls were set to 100 % (absolute mean frequency: 0.03%). Mean values and SEM from 3 measurements per individual and pooling measurements from three wild-type controls are shown.
